# Evaluating Non-Market Values of Agroecological and Socio-Cultural Benefits of Diversified Cropping Systems

**DOI:** 10.1007/s00267-021-01437-2

**Published:** 2021-02-10

**Authors:** Terhi Latvala, Kristiina Regina, Heikki Lehtonen

**Affiliations:** 1 Natural Resources Institute Finland, PL 2, 00791 Helsinki, Finland; 2 Natural Resources Institute Finland, Tietotie 4, 31600 Jokioinen, Finland

**Keywords:** Diverse farming system, Agroecosystem services, Valuation, Sustainable agriculture

## Abstract

We explored how consumers value the ecological and socio-cultural benefits of diversified food production systems in Finland. We used a stated preference method and contingent valuation to quantify consumers’ willingness to pay (WTP) for the benefits of increased farm and regional scale diversity of cultivation practices and crop rotations. Three valuation scenarios were presented to a representative sample of consumers: the first one focused on agroecosystem services on cropland, the second on wider socio-cultural effects and the third was a combination of them. The results suggest that consumers are willing to pay on the average €228 per household annually for the suggested diversification. This is equal to €245 per hectare of cultivated cropland. The results also indicate that 21% of consumers were not willing to pay anything to support more diverse cropping systems. The relatively high WTP for both agroecological and socio-cultural benefits provide important messages for actors in the food chain and for policy makers on future targeting of economic resources within agri-environmental schemes.

## Introduction

Biodiversity and various other ecosystem services are considered valuable for societies all over the world (PBL 2014). In agriculture, provision of ecosystem services is promoted by agri-environmental schemes (AES), e.g., in Europe and in the United States (PBL 2014). Application of agri-environment programmes has been compulsory for EU countries in the framework of their rural development programmes since 1992, whereas they remain optional for farmers. Expenditure on agri-environment measures through rural development programmes totalled ~€25 billion in the EU during 2014–2020 (Eur-Lex 2013). AES implemented in the EU compensate farmers, e.g., for reducing land use intensity and maintaining or introducing biodiversity-rich habitats (Eur-Lex 2013; Science for Environment Policy 2017).

Despite the AES, highly specialized agricultural production regions with crop species monocultures are common in Europe and North America putting the sustainability of the productivity growth at risk (PBL 2014). Monocultures are strongly linked to biodiversity loss globally (Foley et al. 2005; IPES-Food 2016) as well as in northern Europe (Salonen et al. 2007; Tiainen et al. 2020). High-input practices, often connected to monocultures, have been found to cause soil degradation and nutrient leaching to water bodies, affecting negatively ecosystems such as rivers or lakes (Tilman et al. 2002). Nutrient leaching to watercourses has been found to be related to cereal monocultures also in Finland (Manninen et al. 2018; Yli-Viikari 2019). Soil organic matter is gradually decreasing in Finnish croplands (Heikkinen 2019), a development partially explained by the change towards increasing proportion of annual cropping during the last decades (Heikkinen et al. 2013). Cereal and even cereal species monocultures dominate in large parts of southern Finland despite many alternative crops available for diversification of monocultures (Peltonen-Sainio et al. 2017). Area under protein crops, oilseeds, potatoes, sugar beets and other crops is relatively small in south-east part of Finland (OSF 2020a) due to limited domestic demand and excessive imports of protein feed for livestock (OSF 2018). Grass forage crops have been decreasing in terms of cultivation area in southern Finland due to intensification of the dairy sector and concentration of production in certain regions of the country (OSF 2019a, 2020a). Such developments, which are not easily reversed, are common in major dairy producing regions of the European Union, North America, Australia and New Zealand (Clay et al. 2020).

A growing literature suggests that non-market values of impacts of food production on, e.g., water quality, C sequestration, biodiversity, pollution, erosion or GHG emissions are significant and they may even be compared to the market value of agricultural production (Sandhu et al. 2008).

Despite the obvious importance of agroecological ecosystem services, their total value is not currently included in the prices of food and agricultural products. There are few studies in Finland focusing on the non-market value of the agroecological ecosystem services (Grammatikopoulou et al. 2013; Pouta et al. 2014; Tienhaara et al. 2020) and none of them is specifically targeted to the benefits of cropping diversification although it is a key provider of ecosystem services.

This study focuses on the value of diversified cropping in southern Finland, the prime crop production region in the country. We used a stated preference method, contingent valuation, to explore how much consumers would be willing to pay for a broad range of consequences of shifting current monocultures to more diverse cropping systems with a specific emphasis on milk and cheese production. The aims of the study were (1) to elucidate how consumers value agroecosystem services enabled by cropping diversification and (2) to provide consumer perspectives for developing future agricultural and food policies to better support cropping diversification.

We find that the non-market value of diversification benefits is very significant and comparable to the market revenues of crop production in Finland. However, the variability is high in the willingness to pay (WTP). A significant minority of consumers is not willing to pay anything for diversification. We contribute also in showing what is the role of agroecological and socio-economic dimensions of the ecosystem services linked to diversification. All these aspects are important to be considered by policy makers and actors in agricultural value chains. Our results suggest that promoting cropping diversification requires skill due to the variability of consumers’ preferences and opinions. The next section provides more basis for our study, before the methods and results are presented.

## Scientific Background for the Valuation Scenarios

Diversified farming practices under low-input and organic systems sustain and supply multiple agroecosystem services, thus reducing environmental externalities and the need for off-farm inputs. Diverse crop rotations can improve the resilience of cropping systems to multiple environmental stresses and thus increase yield stability and the overall sustainability of food production (Gaudin et al. 2015). Management options that reduce soil disturbances and lengthen the period of soil cover conserve soil carbon (Stockmann et al. 2013) and reduce losses to the watercourses (Valkama et al. 2015; Mhazo et al. 2016). Low-input or diversified farming increases the richness and abundance of species in agroecosystems (Bengtsson et al. 2005; Santangeli et al. 2019). Kremen and Miles (2012) compared biologically diversified and conventional farming systems and identified ten agroecosystem services relevant for the sustainability of food production (Table 1).

**Table 1:** Agroecosystem services of diversified farming systems (Kremer and Miles 2012)

Biodiversity	Pollination services
Soil quality	Carbon sequestration
Nutrient management	Resistance and resilience to climate change
Water-holding capacity	Control of weeds, diseases and pests
Crop productivity	Energy-use efficiency and reduction of warming potential

Cropping diversification has gained attention when strategies towards more sustainable and climate resilient agriculture have been outlined (Soussana et al. 2012). Since cropping diversification can reduce the intensive usage of pesticides and synthetic fertilizers, and mitigate greenhouse gas emissions, it can be seen as one important element of sustainable intensification of agriculture, a key concept and strategy for meeting the challenge of feeding increasing global population (Tilman et al. 2011). Monocultural rotations can be broken, e.g., by winter crops, catch crops, pulses, oilseed crops and clover grass or other grass leys. Diversification of rotations can be combined with diverse management practices like reduced tillage or no-till providing increased biodiversity above and below ground, improved soil quality, enhanced water-holding capacity and increased carbon content of the topsoil thus improving resilience and increased yield stability (Kremen and Miles 2012). Diversification may include low-input management practices with reduced use of synthetic fertilizers, pesticides, machinery, energy and water, with no or little decrease in crop yields (Lin 2011; Smith et al. 2008).

In addition to the farm and field scale diversification, diversification can be considered in regional scale. Although dairy production has had a tendency of gradual concentration to relatively few regions (OSF 2019a), spread of grassland-based dairy production across the country would be an effective way to maintain rotational grasslands and thus diverse crop rotations in a northern country with limited possibilities for extending the vegetated period of fields with other means (Peltonen-Sainio et al. 2017). For example, local farm-scale cheese production with differentiated special products supports this goal and promotes the vitality of local food traditions.

In the following, we outline the building blocks of our valuation scenarios: the agroecological and socio-cultural aspects of food production in Finland linked to the diversification of cropping systems.

### Greenhouse Gases

Agricultural soils produce 70% of Finland’s nitrous oxide emissions (OSF 2019b). Emissions arise especially outside the growing season (Maljanen et al. 2001). Wintertime vegetation cover on fields can decrease nitrous oxide emissions even by one third (Regina et al. 2013).

### Carbon Balance of Soils

According to recent research the organic carbon content of cropland soils decreases with the prevailing agricultural management (Heikkinen 2019). Cropland management with perennial grasses or other deep-rooted species builds up organic matter in soil (Francaviglia et al. 2019; Poeplau and Don 2015; Stockmann et al. 2013).

### Nutrient Leakage

Eutrophication is a major problem for the Baltic Sea and fresh waters. This is a very well-known and visible environmental problem to consumers. Agriculture is a significant source of nitrogen to the environment (Hellsten et al. 2019). In spring cereal production, non-legume catch crops represent an effective method for reducing nitrogen leaching across the varieties of soils and weather conditions in the Nordic countries. Catch crop reduces field nitrogen leaching losses on the average by 50% (Valkama et al. 2015). Based on recent land use data on the feasible crop rotations, it can be estimated that the current area of cover crops can be even tripled (OSF 2020a).

### Better Growing Conditions and Robust Crop Yield

Climate change is expected to increase the risk of plant diseases and pests as well as sensitivity to exceptional weather conditions and weaken growth conditions in Nordic agriculture (Wiréhn 2018). Diverse cultivation can decrease the emergence of pests and diseases and improve the robustness of crop yields in extreme weather conditions. Soil structure and growth conditions are expected to improve due to diversification (Kremen and Miles 2012).

### Abundance and Diversity of Organisms in Fields and Soils

Arable farms often have simple rotations (cultivation of 2–3 plant species, which may also be rather similar, e.g., spring cereals) or monocultures (Peltonen-Sainio et al. 2017). The amount of wildlife organisms and species, especially that of farmland birds and insect, is currently decreasing, e.g., because of declining number of grazing animals (Santangeli et al. 2019; OSF 2019a). Consequently, land use has shifted towards cereals instead of grasslands and leguminous crops (OSF 2020a). Diversified cultivation, reduced tillage and increased number of grazed grasslands increase the variety of flora and fauna, e.g., the number of plant and animal species in fields and soils (Ekroos et al. 2019; Nieminen et al. 2011; Tiainen et al. 2020).

### Organic Dairy Production

The share of organic milk production is about 3% and organic crop production covers 11% of the total arable area in Finland (Finnish Food Information 2019). Organic dairy production features not only organic fertilization or low-input forage crop management; it is also connected to diverse crop rotations and better animal welfare. Organic dairy production offers a bundle of agroecosystem services, and as grazing is obligatory it also provides sceneries with grazing animals.

### Low-Input Production

In low-input production, farms use fewer inputs purchased outside the farm, such as fertilizers, energy, plant protection products and feed. Fostering agrobiodiversity reduces the need for off-farm inputs. Legumes cultivated with grains reduce the use of fertilizers and enhanced floral diversity may attract natural enemies to crops and provide pest control (Kremen and Miles 2012).

### Grazing Animals in Landscape

Dairy and beef farms have been decreasing at a relatively rapid rate, 6–8% per year in southern Finland during the last 30 years (OSF 2019a) and due to that grazing animals are increasingly rare in the landscape. The other reason for reduced presence of grazing dairy cows is that significance of pasture feeding is decreasing. While grazing has earlier been a practice in almost all dairy and beef producing farms, recent information shows that 72% of dairy farms utilize summertime grazing (Finnish Food Information 2019).

### Variation of Crops in the Landscape

Intensive crop production features little or no variation in the landscape as variety of few crop species is low or the same crop is grown on the same site year after year (Peltonen-Sainio et al. 2017). In a diversified cropping system clover and pea species add variation in plant biodiversity, and oilseed rape cultivation brings yellow colour to the landscape.

### Regional Equality

The decrease in the number of dairy farms and dairy cows has been relatively fast in southern Finland compared to other regions (OSF 2019a). Activities like viable local cheese production support the vitality of milk production also in the southern part of the country.

### Rural Jobs

Dairy milk products from raw milk are increasingly produced in relatively few and large specialized dairy processing plants. Approximately 50% of cheese consumed in Finland is imported and the market share of domestically and locally produced cheese has gradually decreased since 2000 (OSF 2018). Domestic cheese production supports the maintenance of jobs in the cheese production chains.

### Tradition of Cheese Processing

Milk products in general and especially cheese, as well as local products, have strong appreciation in Finnish food culture. In 2018, cheese consumption per capita was 26 kg per year (~0.5 kg per week per person) (Luke 2019a). Maintenance of cheese-making skills, knowhow and traditions in various regions of the country supports the cultural heritage of food culture.

In the following section we explain how the value scenarios were built based on the 12 characteristics described above.

## Methods

### Scenario Design

Since most ecosystem services provided by agroecosystems are not exchanged and priced in the market their value can be estimated through a hypothetical market that is presented to respondents of a survey as scenarios. Three different scenarios were illustrated to the respondents in our survey. We presented the scenarios with short explanations of agroecological and socio-cultural changes when cultivation is shifted from monocropping, based on conventional practices, to a more diversified cultivation system. For that, short introductions were given, e.g.: “Crop production can be diversified by changing cultivation practices. Monocropping with conventional tillage can be shifted to cultivation of various crops in the same field, increasing vegetation cover during wintertime and reducing soil tillage, resulting in better growth conditions and soil structure. Cultivation practices affect greenhouse gas emissions and runoff. Moving towards more diverse cultivation techniques increases cost of cultivation”.

Half of the respondents evaluated ecological benefits first (see Table 2) and the other half evaluated socio-cultural benefits first (see Table 3). This was made to avoid an order effect of the valuation task. Finally, scenario 3 combined these two scenarios offering totally 12 attributes (summarizing 5 and 7 attributes). We used colours and visual symbols to make the scenario more visually inviting; orange text in the middle highlighted the current situation and the changes in ecosystem services were highlighted with visual symbols and green colour.

**Table 2:** Agroecological benefits: description of the current status and potential changes

Attribute	Description of current status	Description of change
Greenhouse gases	Fields produce 70% of Finland’s nitrous oxide emissions. Emissions arise especially outside the growing season.	Wintertime vegetation cover on fields decreases greenhouse gas emissions even by 1/3.
Adaptation to climate change	Great susceptibility to diseases and pests and sensitivity to exceptional weather conditions. Weakened soil quality.	Emergence of pests and diseases decreases and robustness of crop yields in extreme weather conditions improves. Soil structure and quality improves.
Soil carbon	The stock of soil carbon in the field has fallen slightly.	Soil organic carbon content increases with less frequent tillage and increased deep-rooted crops in crop rotation.
Runoff leakages	About half of the nutrient leaching to the watercourses comes from the fields. In the current situation, about 9% of the arable land is cultivated with catch and cover crops.	Catch crop reduces field nitrogen leaching by 50%. Catch and cover crop areas can be tripled from current levels.
Abundance and diversity of organisms in croplands	Decreased populations of wildlife organisms and species.	Cropping diversification increases the abundance and diversity of plants and animals in fields and soils.

**Table 3:** Socio-cultural benefits: description of current status and potential changes

Attribute	Description of current status	Description of change
Organic production	The share of organic milk production is about 3%. Organic crop production covers 11% of the total arable area in Finland.	The share of organic milk and crops is increasing.
Low-input production	Fertilizers and feeds bought outside the farm are used in abundance. Cows are mainly high yielding. Limited diversity of crop rotations.	Cow feed is produced using less inputs purchased outside the farm, such as fertilizers, plant protection products and purchased feed. Cows are not necessarily high yielding.
Grazing cows in the landscape	Grazing cows are rarely seen.	Grazing cows are in sight in the landscape.
Crop diversification	Large fields areas are in monotonous cultivation with only few crop species present or with the same crop grown on the same site year after year.	Arable landscape is more diverse and lively. Clover and pea species add variation in plant biodiversity, and oilseed rape brings yellow colour to the landscape.
Regional equality	Milk production has concentrated in the middle and northern part of the country. Milk processing is done in large units.	Local cheese production supports the survival of milk production also in the southern part of the country. Small cheese factories are economically viable.
Rural jobs	In Finland, there are a few dozen small-scale cheese processing factories, which usually are located in rural areas.	Cheese is made by domestic, small-scale cheese companies. Cheese making offers jobs in rural areas.
Tradition	Cheese and local products have a strong role in the Finnish food culture.	Cheese-making skills, knowhow and traditions are maintained in different parts of the country.

### Contingent Valuation Method

We used a stated preference method, contingent valuation (Carson 2000), to estimate the value consumers address to the benefits of shifting from monoculture to diversified cropping systems. This method allowed us to attach non-market benefits of ecosystem services as public good and to attach these benefits to the extra cost of food expenditures. Using the choice experiment method was not possible in our case as it allows only limited number of attributes and levels to be estimated and our study included a total of twelve attributes.

Numerous instruments have been developed to mitigate hypothetical bias in contingent valuation and other stated preference methods. One way of reducing hypothetical bias is the so-called “cheap talk”, where participants are asked to consider about the phenomena of hypothetical bias prior to the valuation question (Lusk 2003). In our study, the respondents were given a short explanation before the valuation question: “Please evaluate as realistically as possible the maximum amount of incremental payment in your monthly food expenditures. It is important that you do not overestimate or underestimate it. Please consider carefully how this incremental cost will affect the monthly expenditures of your household so that you are totally sure that you are willing to pay the sum that is your choice in the question”. To avoid the “order effect” in responses, we split the sample and valued separately ecological and socio-cultural benefit in different order. In order not to overestimate the values, we tested the limits of a reasonable bid vector in a pilot phase (see below in “Sample Properties”) and finally the lower probabilities of the payment interval were use in the analysis phase.

### Ambivalence and Scope Insensitivity

Valuation of non-market goods is not an easy task for consumers. Ambivalence occurs when an individual is forced to make difficult trade-offs; in our case a respondent may have difficulties in resolving ambivalence over trade-offs between monetary value and agroecosystem services. The respondent may have little experience in similar trade-offs and they may have difficulties to identify their indifference between monetary values and environmental amenities (Ready et al. 1995).

We applied a payment vehicle format that allowed expressing uncertainty during the valuation task and this is again one way to mitigate hypothetical bias. Allowing this type of uncertainty for the respondent may ease the pressure of saying “yes” and then avoiding too high WTP estimates. In this study, multiple-bounded dichotomous choice (MBDC) format was used because it allows the respondent to express his/her ambivalence (Welsh and Poe 1998). Respondents were given an identical set of bids and for each bid (e.g., “How surely you would pay maximum 10 cents per month if the described diversification were realized”, “How surely you would pay 50 cents…”, “How surely you would pay 1 euro…”, etc.) they had five response categories to choose from “definitely I would pay”, “possibly I would pay”, “cannot say”, “possibly I would not pay” and “definitely I would not pay”. This question type allows them to express ambivalence in their WTP separately for each bid. Vossler et al. (2003) presented the findings that the proportion of MBDC “definitely would pay” and “probably would pay” responses are good predictors of actual contributions for a good.

Sensitivity to scope or embedding effect refers to a phenomenon in which a wide range of variation is found to occur in WTP for the same good depending on whether the good is valued on its own or valued as a part of a more inclusive package (Kahneman and Knetsch 1992; Veisten et al. 2004). In our case, we were asking the respondents to elicit their WTP in three scenarios with the last one being the sum of the two previous scenarios. If the scope effect is present, values of the more inclusive scenario (combined scenarios 1 and 2) differ from the sum of values in separate scenarios (scenarios 1 and 2).

### Estimation of WTP

The WTP of the respondents can also be estimated non-parametrically, without assuming a utility function or distribution of an error term. In such cases, WTP is estimated using a bid vector, and point estimations of WTP probabilities. According to the economic theory, the proportion of observed “no” responses to each bid should increase when the offered bid (price or cost) increases, yielding genuinely monotonic distribution functions. Sometimes this is not true due to randomness. In that case a Turnbull (1976) distribution-free estimator can be applied (Haab and McConnell 2002).

In the non-parametric estimation of a dichotomic WTP question, the relative proportion of “no” responses was calculated for each bid, a point estimator for the WTP function was made for the each bid ***t***_*i*_ and the relative proportion of “no” responses ***F***_*j*_ was calculated as follows:

1 $$F_j = \frac{{N_j}}{{T_j}},J = 0 \to J$$

where ***N***_*j*_ is the proportion of “no” responses of the combined total ***T***_*j*_ of all “yes” and “no” responses.

WTP was calculated from a monotonic WTP curve by dividing WTP in subranges {***o*** − ***t***_1_, ***t***_1_ − ***t***_2_, …, ***t***_M*_ − ***U***}. To calculate lower bound estimate (LB) of WTP, WTP_LB_ (indicates that the accumulation of the probability mass was calculated only at the lower end bound of the subrange yielding conservative estimates), *F* (0) = 0 (cumulative density function at the LB of WTP) and the upper bound for the WTP must be determined. By using these subranges, WTP was calculated with the following formula:

2 $$E_{\rm{LB}}\left( {\rm{WTP}} \right) = {\sum\limits_{j = 0}^{M^ \ast }} {t_j \times f_{j + 1}^ \ast }$$

where *t*_*j*_ was the offered extra cost and M^*^ was the number of bids.

### Sample Properties

Data sets were collected by a market research company (Makery Ltd). Respondents were contacted by mail and they were remunerated after finishing the questionnaire. Totally 4300 invitations were sent and about 18% of the receivers started the questionnaire, and 4% of them did not finish the questionnaires. During data quality checking, totally 20 respondents were discarded either due to a too fast response time or due to no variation in their responses.

A pilot study (*n* = 100) was conducted in December 2018. The total of 600 responses to the final questionnaire was collected in January 2019. This was implemented through an online questionnaire to a representative sample of the adult-aged (18 years old or older) Finnish population. The sample was selected by three criteria: sex, age and residence. By using these criteria also the education level and income classes represented Finnish population well (Table 4). There was a control in the internet-based survey ensuring that the samples of respondents answering in the two different versions of the survey form were similar with respect to age, sex, income and residence.

**Table 4:** Characteristics of the sample compared to the population of Finland

	Sample	Finland
Sociodemographic information		
Age of people over 18 years old		
18–24 years (%)	10.8	9.9
25–34 years (%)	16.0	15.8
35–44 years (%)	15.1	15.7
45–54 years (%)	16.9	15.3
55–64	17.2	16.4
65+	24.1	27.0
Gender (% women)	51.0	51.0
Household income (€/year before taxes)		
<€10,000 (%)	5.8	18.4^a^
€10,001–€20,000 (%)	10.7	22.1
€20,001–€40,000 (%)	23.5	36.3
€40,001–€60,000 (%)	19.6	14.8
€60,001–€80,000 (%)	13.9	13.9
€800,001–€120,000 (%)	7.9	N.A.^b^
>€120,001 (%)	1.5	N.A.^b^
Educational level (%)		
Lower education (basic education)	10.2	27.9
Upper secondary education	51.5	40.3
Higher education	37.9	31.0
Other	0.8	0.8

^a^In our data, only responses from over 18 years old were included, while income statistics includes low incomes of younger people

^b^€80,000–€99,999: 1.8%; €100,000–€150,000: 1.3%; >€150,000: 0.7%

Source: OSF 2020b

## Results

### Unwillingness to Pay

The results indicate that 21% (*n* = 126) of consumers were not willing to pay any extra expenditure to support more diverse cropping systems. Almost half of these respondents indicated that they cannot afford to pay more (*n* = 58, 46% of no responses). The statement “Consumers or taxpayers should not pay extra cost” was agreed by 31% of the respondents that were not willing to pay any extra. Thirty per cent of the respondents in this group also stated that the current cultivation practices are diverse enough.

### Opinions of Consumers on the Importance of Agroecosystem Services

Opinions of consumers on the significance of different aspects of Finnish agriculture, including the effects that are implications of diversified cropping systems, were collected by using a five-point Likert-scale, from 1 (very small) to 5 (very high). The opinions on the importance of diversification and other agroecosystem services related to cheese making are shown in Table 5.

**Table 5:** Importance of different aspects of diversification from the viewpoint of consumers

	Mean—scale 1–5 (standard deviation in parenthesis)	Cannot say (% of respondents)
1. Domestic food production and processing^a,b^	4.41 (0.82)	1.8
2. Nutrient leakages from agriculture will decrease^c^	4.32 (0.88)	2.8
3. Finnish food culture is preserved (tradition, knowledge and processing skills)^b^	4.25 (0.90)	1.9
4. The ability of fields to act as carbon sink and combat climate change is improving^c^	4.18 (0.89)	5.0
5. The jobs in rural areas remain^b^	4.13 (0.94)	2.7
6. Greenhouse gas emissions from agriculture are decreasing^c^	4.12 (0.96)	3.3
7. Abundance of wildlife organisms in the field and soil^c^	4.09 (0.87)	5.9
8. Diversity of arable crops^a,c^	4.06 (0.85)	3.9
9. The growing conditions in the fields and robustness of crop yields under varying conditions improve^c^	4.04 (0.86)	6.7
10. Organic production is becoming more common^c,b^	3.87 (1.05)	4.0
11. The arable landscape has varied vegetation^b^	3.84 (0.93)	5.9
12. The variety of species of production animals^a,c^	3.78 (0.96)	5.6
13. The grazing cows are in sight in the landscape^b^	3.72 (1.05)	3.1
14. Low-input production methods become more common (less inputs from outside the farm)^b^	3.61 (0.97)	15.2
15. Evenness of regional distribution of agricultural production and processing^b^	3.54 (0.99)	9.5

^a^Not solely included to the valuation scenario

^b^Indicating socio-cultural benefits

^c^Indicating ecological benefits

Totally 15 aspects were identified, but only 12 were selected to the final valuation scenario. Among the highest ranked were domestic food production, followed by features arising from diversified cropping (decreasing nutrient leakages, preserving Finnish food culture, carbon sequestration and rural jobs). Low-input production was found difficult to estimate since 15% of respondents answered, “cannot say”.

### Willingness to Pay for Agroecosystem Services

For the respondents who were willing to pay for more diverse cropping systems (79%, *n* = 474), we estimated mean WTP for the three scenarios. The ranges of WTP to the offered bids were estimated from responses “definitely would pay”, “possibly would pay” and “definitely would not pay” (Table 6). It seems that scenario 1 (ecological benefits), with the effect coming from diversified cropping system, was marginally higher valuated (WTP €16/month/household) than scenario 2 with socio-cultural benefits (WTP €15/month/household). Responses of the group “possibly would pay” resulted in a higher value for the socio-cultural benefits. Finally, the third scenario combining scenarios 1 and 2 had a relatively small (19–27%) increase (considering the increase in the number of attributes by close to 100%) on WTP compared to that in scenario 1 or 2. Still the third scenario had a higher value since it had all attributes included that were present in scenarios 1 and 2. This result is consistent with the decreasing marginal utility from additional benefits. It is also important to see high variability in the WTP as indicated by the average WTPs and their certainty intervals based on “possibly would pay”, “definitely would pay” and “definitely would not pay” answers, i.e., many individuals were willing to pay significantly more (or less) than the average WTP in case “definitely would pay” (Table 6).

**Table 6:** Estimated mean WTP in three scenarios (€ per household per month or year)

	WTP for ecological benefits (scenario 1) € per month [95% CI]	WTP for socio-cultural benefits (scenario 2) € per month [95% CI]	WTP for combined scenario (scenario 3) € per month [95% CI]	WTP for combined scenario (scenario 3) € per year
Mean Definitely would pay	16 [13, 19]	15 [12, 18]	19 [15, 23]	228
Mean Possibly would pay	32 [27, 37]	35 [29,41]	40 [32,48]	480
Mean Definitely would not pay	85 [75, 95]	121 [111, 131]	124 [104, 144]	1488

## Discussion

The results of the survey suggest that consumers value several benefits of crop diversification. As much as 79% of Finnish consumers were willing to pay higher food expenses for diversified cropping indicating that positive socio-cultural and ecological effects of crop diversification such as domestic food production, food security, nutrient leaching, food culture or carbon sink are very significant for consumers.

Results highlight some favourable aspects of organic production such as increased cropping diversification, improved and more robust crop growing conditions in the fields and increased landscape diversity. However, regional distribution of agricultural production and processing within Finland, and low-input production methods, and less purchased inputs on farms as a consequence of diversification were considered relatively least valuable. Many consumers could not say if the low-input production methods are valuable. This is understandable since low-input production methods are related to the farm level management consumers are most often not aware of. Most consumers thus cannot evaluate the meaning and significance of the changes in, e.g., input use at the farm level.

In the first scenario valuation concerns the ecological benefits of crop diversification and in the other scenario the perceived socio-cultural benefits of local and organic cheese production. Merging scenarios 1 and 2 in scenario 3 resulted in a relatively small 19–27% increase in WTP for scenario 3 than the WTP for scenario 1 or 2. This suggests that ambivalence is present in ecological valuation studies and it has an effect on consumers’ estimates regarding ecological and socio-cultural benefits of diversified cropping system. Therefore, it is important to take ambivalence into account when designing a payment vehicle for a survey. As Venkatachalam (2004) concludes, embedding is an ordinary economic phenomenon that not only occurs in public goods, such as ecosystem services, but also in private market goods. One of the reasons why embedding shows up in contingent valuation studies having many consecutive scenarios is due to a simple economic theory: from the marginal utility theory we can deduce that the utility of an individual at a margin declines for the subsequent bundle of commodity they consume (Hanemann 1994). Our results show decreasing marginal utility of consumers concerning the benefits of cropping diversification even if our methods did not assume any utility function or distribution.

Since hypothetical valuation methods do not deal with real money they are not incentive compatible, WTP values elicited in the third scenario can be considered more conservative and realistic choice for further analysis. Hence we recommend using the WTP estimates for ecological and socio-cultural benefits of the third scenario in future studies.

When aggregating results from the responses “definitely would pay” in relation to Finnish agricultural sector, the calculated non-market value of diversified cropping can be calculated as follows: the WTP per household, €228, multiplied by the number of households (the questions were asked on per household basis in the survey), and divided by the area cultivated (1.996 million ha under agricultural crop production in Finland 2017; Luke 2019b) yields €245/ha per year in Finland. This estimated value of cropping diversification, €245/ha, can be considered a very significant value since average cereal yield (3.5 tons of crop yield per ha, 2000–2014 average price of barley ~€150/ton) gives €525/ha market revenue for a crop farm. The value of €245/ha per year can be also compared to the farm subsidies paid per ha of organic crop production: €160/ha. However, our estimate €245/ha is clearly smaller than the annual total market revenue in agriculture (€1600/ha) at the national level in Finland, considering also livestock production (Luke 2019c). Hence the value of diversified cropping can be as high as 47% compared to the annual market revenues at cereals, and 15% compared to the total market revenues in agriculture in Finland.

Vossler et al. (2003) suggest that the average WTP from “possibly would pay” responses are the closest on real WTP when using MBCD in measuring WTP, as we also did. Results from “possibly would pay” responses average to €480/household in our study. This is ~€1 billion per year at the whole country level with ~2 million households. Following the reasoning of Vossler et al. (2003) means approximately doubling the calculated “definitely would pay” values, yielding €516/ha per hectare. This is close to the average value of market revenues in barley production, and as much as 32% of the total market value of agricultural production in Finland. However, we see that this high value (€516/ha) may not be fully warranted based on our results since hypothetical valuation methods such as contingent valuation do not deal with real money and are not incentive compatible. Hence, we see the results from “definitely would pay” responses as a conservative and realistic “lower bound” for the non-market value of diversified cropping, with a possibility that the real value is higher.

Nevertheless, our results fit into the ranges estimated by Sandhu et al. (2008). Their estimate for the non-market benefits ranged from US$50 to US$1240 per hectare per year for conventional fields and from US$460 to US$5240 per hectare per year for organic fields. A recent European study of Alcon et al. (2020) evaluated the non-market value of cropping diversification in fruit tree production in Murcia region in Spain using a similar kind of consumer survey but based on a choice experiment approach. However, the survey of Alcon et al. (2020) was narrower and did not include as many attributes as our study, e.g., food tradition or rural jobs. Mean WTP analysis shows that, on the average, respondents are willing to pay a total amount of €24.58/household/month in order to support diversified cropping. This is higher than the WTP calculated in our study. Furthermore, Alcon et al. (2020) calculated non-market value of high-efficient irrigated intercropping system as high as €1361.6/ha/year. This is potentially even higher than the crop financial benefits, e.g. in some cases of low profitable farmlands, such as almond crops. These results are not strictly comparable since Bernués et al. (2014) showed that non-market values measured by using local sample resulted higher value than national sample. This is another reason we rather consider the WTP estimate based on “definitely would pay” as a conservative and being main result of our study.

However, rather than only emphasizing the relatively large potential non-market values calculated for cropping diversification, which seems to be much dependent on the national and local context and the value of primary agricultural production in specific production lines, it is also important to see that in our study 21% of respondents were not willing to pay anything for increased cropping diversity in their food expenditures. Almost half of them (46%) expressed a view that they cannot afford to pay more for food. Moreover, 30% of those not willing to pay agreed with a view that consumers should not be the ones who pay for the diversified cropping, and 31% agreed with a view that current cropping practices are diversified enough. Thus, the diverse views of consumers and underlying arguments behind the views are worth to be considered when planning future agri-environmental or other policies directing tax funds to cropping diversification. Questions like “Who should really pay for the non-market values related to cropping diversification and why” are indeed valid for policy planners, farmers and other actors in the food and agriculture values chains. They should define who are the customers and beneficiaries of the agri-ecosystem services, and what is the fair rate and method of payment if part of the values is not included in the food prices.

## Conclusions

Our results show that 79% of households were willing to pay extra for cropping diversification and that this corresponds to a significant monetary value of ecosystem services. The calculated total non-market value of cropping diversification at the country level can be as high as 47–95% compared to the annual market revenues of cereals, and 15–32% compared to the total market revenues in agriculture in Finland. Several other similar but not identical studies and non-market valuations also show significant or even higher WTP results compared to our study. However, we see this kind of studies and their results somewhat specific to countries and cases of diversifications and one should be careful in making general conclusions.

Nevertheless, better understanding of the consumer point of view is important in finding both market and policy-based solutions for diversification. The effectiveness of agroecological schemes needs to be developed further but a higher contribution by consumers can also likely be anticipated to fund future transition towards more sustainable food production.

Findings on the valuation of different ecosystem services help different actors of the food chain or policy makers to stress the most valued consequences and use the related arguments when, e.g., motivating the use of public expenditures. This study showed that positive societal implications of cropping diversification were valued slightly higher than direct field level effects of diversification. In particular, improved maintenance of domestic food production and processing, reduced nutrient runoffs from agriculture, maintained food culture and tradition, as well as improved carbon balance of agriculture and the number of jobs in rural areas were valued high. Rather traditional arguments based on ecology have been used in promoting, for example, organic and low-input agriculture but their effects on carbon sequestration and more resilient food production with positive effects on rural jobs and local food culture have been less emphasized. Using a larger selection of arguments would help to reach a wider variety of consumer types.

## Data Availability

The data set analyzed during the study, ensuring the anonymity of the respondents, is available from the corresponding author upon reasonable request after the completion of the project.
